# Prevalence and Associations of Poor Sleep in Patients with Relapsing-Remitting Multiple Sclerosis on Disease-Modifying Therapy

**DOI:** 10.3390/jcm14217837

**Published:** 2025-11-04

**Authors:** Dimitar Taskov, Sonya Ivanova, Nikolay Topalov, Alexandra Barkalova-Atanasova, Nikolay Yordanov, Mitko Yurukov, Karina Atanasova-Ivanova, Paulina Ilieva-Nedeva, Antonia Nikolova, Sonia Chipeva, Ivan Milanov

**Affiliations:** 1 Multiprofile Hospital for Treatment in Neurology and Psychiatry, St. Naum, 1113 Sofia, Bulgaria; 2 Department of Neurology, Medical University, 1431 Sofia, Bulgaria; 3 Department of Statistics and Econometrics, University of National and World Economy, 1700 Sofia, Bulgaria

**Keywords:** multiple sclerosis, poor sleep, insomnia, anxiety

## Abstract

**Background:** Sleep disturbances in the multiple sclerosis (MS) population are increasingly recognized, but the factors driving this association remain understudied. This study aimed to determine the prevalence and associations of poor sleep quality in the relapsing–remitting MS (RRMS) population receiving disease-modifying therapy (DMT). **Methods:** We conducted a cross-sectional study that enrolled 399 individuals diagnosed with RRMS on DMT. Data on patient demographics, clinical presentation, and treatment were systematically evaluated. Sleep-related outcomes were assessed using validated questionnaires—the Pittsburgh Sleep Quality Index (PSQI), Insomnia Severity Index (ISI), Epworth Sleepiness Scale (ESS), Fatigue Severity Scale (FSS), STOP-Bang questionnaire, and Hamilton Anxiety Rating Scale (HAM-A). Independent associations of poor sleep were examined using log-binomial regression to estimate risk ratios (RR). **Results:** Poor sleep was reported in 42% of the participants in our cohort. In multivariable analysis, only insomnia severity (RR = 1.07; 95% CI 1.05–1.09, *p* < 0.001) and anxiety (RR = 1.02; 95% CI 1.01–1.04, *p* = 0.001) remained independently associated with poor sleep. **Conclusions:** Sleep disturbances are common among patients with RRMS. Insomnia severity and anxiety, rather than demographic or disease-related characteristics, showed independent associations with impaired sleep. Routine screening and targeted interventions addressing insomnia and anxiety may improve sleep quality and, consequently, overall quality of life in this population.

## 1. Introduction

Multiple sclerosis (MS) is a chronic autoimmune disease of the central nervous system (CNS) characterized by inflammation, demyelination, axonal injury, and neurodegeneration. MS affects more than 2.8 million people worldwide, and both incidence and prevalence continue to increase. Over the last three decades in Bulgaria, incidence and prevalence have risen from 1.03/100,000 and 44.5/100,000 to 4.2/100,000 and 121.2/100,000, respectively [1,2].

The clinical presentation of MS most commonly involves dysfunction of the visual, sensory, motor, bladder/bowel, cognitive, and coordination systems, reflecting multifocal CNS involvement. The majority of patients are diagnosed with the relapsing–remitting form (RRMS), characterized by clearly defined clinical attacks followed by periods of partial or complete recovery. These manifestations substantially affect both physical and cognitive functioning.

Sleep disturbances are increasingly recognized as a significant clinical concern in MS, affecting up to 74% of patients [3]. Poor sleep has been associated with higher relapse frequency, greater disease severity, and an increased risk of cardiometabolic comorbidities, with consequent reductions in quality of life [4,5,6]. Despite this burden, sleep disorders in MS remain frequently under-recognized and undertreated [7]. Prior studies suggest multiple, often overlapping contributors, including impairment of sleep–wake regulation, secondary symptoms such as pain, spasticity, and nocturia, and comorbid mood disorders [8,9,10].

Data on sleep quality in MS from Central and Eastern Europe, including Bulgaria, remain limited. Healthcare context and cultural factors may shape sleep-related complaints and care pathways in this region. To our knowledge, no prior study has evaluated sleep quality and its associations in patients with RRMS in Bulgaria. The primary aim of this study was to determine the prevalence and associations of poor sleep quality in patients with RRMS receiving DMT. The secondary aim was to summarize the psychometric performance of the sleep-related questionnaires used in this cohort.

## 2. Materials and Methods

### 2.1. Ethics Statement

This study was approved by the local ethical committee and was performed in accordance with the Declaration of Helsinki. All participants provided written informed consent before enrollment.

### 2.2. Study Participants

This cross-sectional observational study included consecutively recruited adult patients with a confirmed diagnosis of MS during their routine visits to the Multiprofile Hospital for Treatment in Neurology and Psychiatry, St. Naum, Sofia, Bulgaria, from April 2024 to March 2025. Diagnoses of MS were confirmed according to the 2017 McDonald criteria by board-certified neurologists at the tertiary center [11]. Inclusion criteria were age ≥ 18 years, a confirmed diagnosis of RRMS, and current treatment with a disease-modifying therapy. Patients with another form of MS, incomplete clinical data, other neurological or psychiatric diseases, a prior diagnosis of sleep disorders, or a relapse in the last 3 months were excluded from the analysis.

### 2.3. Clinical Evaluation

A neurologist experienced in demyelinating diseases evaluated all patients. Clinical and demographic data, including age and sex, were collected. Anthropometric variables included body mass index (BMI). Current neurological disability was assessed using the Expanded Disability Status Scale (EDSS) [12]. Additional information was extracted from electronic medical records, including disease duration, initial clinical presentation (involvement of the visual, pyramidal, sensory, brainstem, and cerebellar functional systems, bowel/bladder symptoms, impaired mobility, and problems with thinking and memory), and EDSS at diagnosis. Data regarding DMT were collected, including efficacy classes: moderate-efficacy (interferon-β (IFN-β) formulations, glatiramer acetate, teriflunomide, and dimethyl fumarate) and high-efficacy (fingolimod, natalizumab, alemtuzumab, ocrelizumab, ofatumumab, and cladribine). We additionally recorded the current DMT, prior DMT exposure (history of more than one DMT), and type of therapy switch: horizontal (between agents of the same efficacy class), vertical (escalation from a moderate-efficacy to high-efficacy agent), or both. We also recorded the duration of DMT exposure and the interval from MS diagnosis to DMT initiation.

### 2.4. Evaluation of Sleep and Related Symptoms

Pittsburgh Sleep Quality Index (PSQI)—19-item instrument of global sleep quality over the past month, summarizing seven components (subjective sleep quality, latency, duration, efficiency, disturbances, use of sleep medication, daytime dysfunction). Component scores (0–3) sum to a global score of 0–21 (higher = worse overall sleep quality). Poor sleepers were defined as PSQI > 5 [13].

Insomnia Severity Index (ISI)—7-item scale quantifying insomnia severity (difficulty initiating/maintaining sleep, early awakening, dissatisfaction, interference with daytime functioning, noticeability, distress) over the past 2 weeks; items 0–4, total 0–28 [14].

Epworth Sleepiness Scale (ESS)—8-item questionnaire assessing the likelihood of accidentally falling asleep in everyday situations. Each item is scored 0–3, with a total score of 0–24. A score > 10 indicates excessive daytime sleepiness [15].

Fatigue Severity Scale (FSS)—9-item scale measuring the impact of fatigue on daily functioning. Items are scored 1–7, giving a total score of 9–63, with ≥36 suggesting significant fatigue [16].

STOP-Bang Questionnaire—8-item screening tool for obstructive sleep apnea, incorporating symptoms (snoring, tiredness, observed apnea, hypertension) and anthropometric factors (BMI, age, neck circumference, sex). Scores range 0–8, with ≥3 indicating increased risk for OSA [17].

Hamilton Anxiety Rating Scale (HAM-A)—clinician-administered, 14-item scale assessing anxiety severity, scored 0–4 per item [18].

The PSQI, ESS, HAM-A, and ISI were provided in their official Bulgarian versions by the MAPI Research Trust (https://eprovide.mapi-trust.org). The Bulgarian version of the FSS was obtained from the copyright holders. The official Bulgarian version of the STOP-Bang questionnaire, provided by the developers, was also used. Because the psychometric properties of Bulgarian versions have not been systematically evaluated, we assessed internal consistency for each instrument using Cronbach’s α, item–total correlations, and “α if item deleted”.

### 2.5. Statistical Analysis

Data were analyzed using SPSS 23.0 for Windows (IBM Corp., Armonk, NY, USA). Statistical significance was defined as two-tailed *p* < 0.05. Continuous variables (age, BMI, current EDSS and EDSS at diagnosis, disease duration, number of relapses, time from diagnosis to DMT initiation, DMT duration, and questionnaire scores—PSQI, ISI, FSS, ESS, HAM-A, STOP-Bang) were summarized as mean ± SD or median (IQR) according to the Shapiro–Wilk test and compared using the independent-samples *t* test or the Mann–Whitney U test, respectively. Categorical variables (sex, initial clinical presentation by functional system, prior DMT exposure, DMT efficacy class, type of therapy switch, current DMT agent) were presented as counts (%) and compared using the χ^2^ test or Fisher’s exact test, as appropriate. The primary outcome was poor sleep (PSQI > 5). Because outcome prevalence was high (~42%), we estimated risk ratios (RR) using log-binomial regression, following established recommendations [19]. When non-convergence occurred, we fitted modified Poisson models with robust (sandwich) standard errors as a prespecified alternative, utilizing the approach proposed by Zou et al. [20]. Candidate predictors with *p* < 0.10 in univariable analyses and clinically relevant covariates were considered for multivariable modeling. Covariates were pre-specified on clinical/causal grounds, and estimates are interpreted as conditional associations given the included covariates. Multicollinearity was assessed using variance inflation factors (VIFs), with values > 5 indicating concern. The events-per-variable (EPV) ratio (target ≥ 10) was monitored for model stability. Correlations among questionnaire scores were examined using Pearson coefficients, and the strength was interpreted using conventional thresholds.

## 3. Results

### 3.1. Demographic and Clinical Characteristics

A total of 399 patients with RRMS were included in the analysis. The median age of the patients was 41 years (IQR 34–49), and 267 of them (66.9%) were female. The median disease duration was 11 years (IQR 5–17), and the median current EDSS score was 3.0 (IQR 2.0–3.5). The most common initial clinical presentations involved the pyramidal (61.7%), cerebellar (59.1%), sensory (49.4%), and visual (33.3%) functional systems, whereas brainstem involvement, bowel/bladder symptoms, mobility impairment, and cognitive dysfunction were less frequently reported by our cohort. In 68.4% of the patients, the clinical presentation included more than one functional system. The median EDSS at diagnosis was 2.0 (IQR 1.5–2.5). At the time of evaluation, 85.2% were receiving moderate-efficacy agents, while 14.8% were on high-efficacy ones. Among moderate-efficacy therapies, dimethyl fumarate was the most commonly used (35.6%), followed by glatiramer acetate (26.6%), interferon beta-1a (13.3%), teriflunomide (6.3%), and peginterferon beta-1a (2.8%). Among high-efficacy therapies, ocrelizumab (6.3%) and fingolimod (5.3%) were most frequently prescribed, followed by ofatumumab (1.5%), natalizumab (1.3%), and cladribine (0.3%). The median time from diagnosis to DMT initiation was 2 years (IQR 1–7). In total, 46.4% of patients received more than one DMT during their course of disease. A therapeutic switch had been performed in 46.1% of patients, with the majority of switches being horizontal (35.8%) (Table 1).

### 3.2. Sleep Quality

The study sample had a median PSQI score of 7 (IQR 5–9). Regarding insomnia severity within the study cohort, the median ISI score was 5 (IQR 1–9). The median ESS score was 5 (IQR 3–8), with 11.5% of patients experiencing excessive daytime sleepiness. The median FSS score was 25 (IQR 16–42), and 34.6% of patients had significant fatigue. The median STOP-Bang score was 1 (IQR 1–2), and 22.8% of the respondents were classified as being at increased risk for obstructive sleep apnea. The median HAM-A score was 11 (IQR 5–17).

### 3.3. Internal Consistency of the Questionnaires

Internal consistency analysis showed Cronbach’s α values ranging from 0.45 for the STOP-Bang to 0.95 for the FSS, indicating acceptable to excellent reliability for the majority of instruments utilized in this study. The PSQI, ISI, ESS, FSS, and HAM-A demonstrated α values above the commonly accepted threshold of 0.70, while the STOP-Bang exhibited poor internal consistency. Item–total correlations were all ≥0.44, except for the STOP-Bang (−0.01–0.36). “Alpha if item deleted” analysis showed minimal improvement for most scales, suggesting stable internal structure. Detailed psychometric characteristics for each instrument are presented in Table 2.

### 3.4. Correlation Analysis Between Questionnaire Scores

Pearson correlation analysis revealed positive associations between PSQI and ISI (r = 0.78, *p* < 0.001) and between FSS and HAM-A (r = 0.66, *p* < 0.001). ISI was also correlated with HAM-A (r = 0.62, *p* < 0.001) and FSS (r = 0.60, *p* < 0.001) (Table 3).

### 3.5. Comparison of Good vs. Poor Sleepers

Out of 399 patients, 167 (42%) were classified as poor sleepers. These patients were older than those with good sleep (*p* = 0.02). No significant differences were observed between the two groups in sex, BMI, EDSS at diagnosis, and current EDSS, disease duration, or number of relapses. Regarding DMT, patients on high-efficacy therapy were more frequently classified as good sleepers compared to those on moderate-efficacy therapy (*p* = 0.03). At the individual-agent level, distributions are reported descriptively and were not subjected to formal hypothesis testing. The duration from diagnosis to treatment initiation and the total duration of treatment did not differ significantly between good and poor sleepers. In terms of sleep- and symptom-related measures, poor sleepers had substantially higher STOP-Bang scores (*p* < 0.001). They reported greater insomnia severity (*p* < 0.001), fatigue (*p* < 0.001), daytime sleepiness (*p* < 0.001), and anxiety levels (*p* < 0.001) compared to good sleepers (Table 4).

### 3.6. Associations with Poor Sleep Quality

In univariable analyses, several demographic, clinical, and questionnaire-derived variables showed associations with poor sleep quality, including higher scores of ISI, HAM-A, FSS, ESS, STOP-Bang, older age, and high-efficacy therapy vs. moderate. When these variables were entered into the multivariable log-binomial model, only ISI (adjusted RR 1.07, 95% CI 1.05–1.09, *p* < 0.001) and HAM-A (adjusted RR 1.02, 95% CI 1.01–1.04, *p* = 0.001) remained independently associated variables (Table 5). Multicollinearity was not a concern (all VIFs < 5), and the events-per-variable ratio (16.7) exceeded the recommended threshold, supporting the stability of the regression estimates.

## 4. Discussion

In this cross-sectional study of 399 patients with RRMS receiving DMT, we found that poor sleep quality was present in 42% of the cohort. To our knowledge, this is the first study to evaluate sleep quality and associations of poor sleep in Bulgarian patients with MS, thereby providing novel evidence from an underrepresented Eastern European population. The prevalence in our sample falls within the middle range of prior reports in RRMS-only cohorts internationally. For example, Laslett et al. reported that 67% of patients had sleep disturbances [21], while a Ukrainian study found 40% with sleep disorders among 105 individuals [22]. Taken together with the Bulgarian cohort, these findings suggest a similar burden across settings and underscore the need for context-specific screening and management pathways in MS services.

The most robust and consistent finding of our study was that insomnia severity and anxiety symptoms were independently associated with poor sleep, while demographic and disease-related factors lost significance. Several studies across different countries have demonstrated that psychiatric symptoms, particularly anxiety and depression, are among the strongest determinants of subjective sleep quality in MS [23,24,25]. A large cohort study reported that anxiety–depression symptom clusters were associated with poorer sleep quality [21]. Interestingly, the previous literature shows that anxiety, more than depression, adversely affects sleep architecture and subjective sleep quality in MS [10].

Another significant finding is that higher insomnia severity was independently associated with poor sleep. Our results align with previous research, showing that higher ISI scores are associated with lower sleep quality in patients with MS [26,27]. Both anxiety and insomnia are known to interact bidirectionally with sleep, heightening arousal, cognitive hypervigilance, and dysregulating stress-axis activity, which may prolong sleep latency, reduce sleep efficiency, and exacerbate daytime symptoms [28]. The correlation we observed between PSQI, ISI, and HAM-A further reinforces this close interdependence. Although PSQI and ISI were strongly correlated, they capture related but distinct constructs: PSQI reflects global sleep quality across multiple domains, whereas ISI quantifies insomnia severity, including symptoms intensity and daytime impact. This conceptual distinction justifies including ISI as a predictor of poor sleep (PSQI > 5) and aligns with prior MS literature highlighting insomnia as a principal correlate of subjective sleep impairment [8,9,10].

Although DMT class, age, initial clinical presentation, STOP-Bang, FSS, and ESS were associated with sleep quality in univariable models, these associations attenuated and lost significance after statistical adjustment (Table 5). This attenuation likely reflects the overlapping influence of insomnia and anxiety measures, as well as shared variance among related symptom domains. Because treatment allocation in MS is influenced by disease activity and other clinical factors, confounding by indication is likely. Without an untreated comparison group, we avoid making causal interpretations regarding DMTs and sleep outcomes. Prior work that adjusted for confounding by indication similarly found no association between DMT class and self-reported sleep quality [29]. In contrast, reports of DMT-related sleep changes typically focused on short-term, peri-administration windows and used objective measures such as actigraphy [30,31].

Importantly, the attenuation observed in multivariable models may act as intermediate variables through which broader MS-related symptom burden is associated with perceived sleep quality. Factors such as anxiety, fatigue, and insomnia severity likely act as mediators through which disease activity and clinical burden influence perceived sleep quality. This interpretation suggests that treating these symptom domains—for example, through anxiety management or cognitive-behavioral therapy for insomnia—could indirectly contribute to improved sleep quality in MS, beyond demographic or treatment-related influences.

In our study, older patients seemed more likely to report poor sleep. However, age did not show an independent association. Prior studies have shown conflicting results, and our findings support more recent evidence that age itself is not a determinant of sleep disturbances in MS [3,32].

In this DMT-treated RRMS cohort, low disability was observed even after 5–17 years of a disease duration. First, the sample comprised only relapsing–remitting patients (progressive phenotypes were excluded) and patients were managed in a tertiary center with sustained DMT exposure. Second, the EDSS is non-linear and weighted towards ambulation and pyramidal signs; common RRMS complaints relevant to sleep (fatigue, anxiety, cognitive and mood symptoms) often do not materially increase EDSS at lower scores. Third, early initiation and maintenance of DMT, together with regular follow-up, may reflect cohort composition and care pathways rather than disease inactivity per se. Taken together, these factors can explain the coexistence of longer disease duration and relatively low current EDSS in our cohort.

In our cohort, the initial clinical presentation of MS did not appear to have a significant association with subsequent sleep quality, consistent with the findings of Khedr et al. [33]. Although sensory involvement, pyramidal dysfunction, and brainstem lesions might be expected to predispose patients to disturbed sleep through chronic pain, nocturnal spasticity, or urinary frequency, our findings indicate that these manifestations are not independently associated with sleep quality [4].

Higher scores of STOP-Bang, FSS, and ESS suggested a link with poor sleep quality, which was not reported in the multivariate analysis. However, correlation analyses revealed positive associations between PSQI and ISI, FSS, and HAM-A, indicating overlapping but distinct contributions of these constructs to the overall burden of poor sleep. There was a weak correlation between STOP-Bang and FSS, which demonstrated limitations of the questionnaire in the MS population as previously mentioned in the literature [34]. Cronbach’s α exceeded 0.70 for PSQI, ISI, ESS, FSS, and HAM-A, whereas STOP-Bang showed lower internal consistency, consistent with prior cautions regarding its use in MS.

The clinical implications of our findings are twofold. First, clinicians should maintain a high index of suspicion for sleep problems in MS patients, particularly when psychiatric symptoms such as anxiety are present. Routine use of validated screening tools can help identify under-recognized sleep disturbances that might otherwise be mistaken for fatigue or disease progression. Second, management strategies should prioritize interventions targeting insomnia and anxiety. For example, Siengsukon et al. demonstrated that cognitive behavioral therapy for insomnia (CBT-I) was more effective than active control conditions in improving sleep quality and treatment of insomnia in MS patients [35].

Strengths of our study include the well-characterized cohort of RRMS patients all receiving DMT, the consecutive recruitment minimizing selection bias, and the comprehensive assessment of sleep, fatigue, daytime sleepiness, and anxiety using validated questionnaires. We also provided internal consistency analyses of the official Bulgarian versions of these scales.

Several limitations should be acknowledged. The cross-sectional design precludes causal inferences regarding the relationship between sleep and MS outcomes, and the absence of a healthy control group limits the ability to determine whether sleep disturbances are directly related to MS or represent common problems seen in the general population. As the study enrolled only DMT-treated RRMS patients and did not include an untreated comparison group, it was not designed to assess causal effects of DMTs on sleep. Consequently, treatment variables were summarized descriptively, and associations were interpreted cautiously due to potential confounding by indication. PSQI is a self-reported instrument and, while widely used, does not capture the full spectrum of objective sleep features (e.g., sleep architecture, periodic limb movements, and respiratory events). Moreover, the conventional threshold PSQI > 5 may not generalize uniformly across populations and clinical contexts. We also did not assess restless legs syndrome or depression, both of which can affect sleep quality. In addition, the lack of objective measures such as polysomnography or actigraphy may have restricted the characterization of sleep disturbances. Future research should employ longitudinal designs and objective assessments to clarify temporal links between sleep, psychiatric comorbidities, and disease progression. The effects of newer DMTs on sleep, as well as the underutilization of sleep disorder treatments and the contribution of comorbidities, also warrant further investigation.

## 5. Conclusions

In conclusion, poor sleep quality was highly prevalent among Bulgarian patients with RRMS, affecting 42% of the cohort. Insomnia severity and anxiety emerged as the strongest independent associations, while demographic, disability, and treatment-related factors did not show a significant relationship. These findings highlight the need for systematic screening and targeted management of sleep and psychiatric symptoms in MS to improve quality of life and functional outcomes.

## Figures and Tables

**Table 1:** Baseline demographic, clinical, and treatment characteristics.

Characteristics	Value, *N* = 399	
Age, median, IQR	41	34–49
Female sex, *n*, %	267	66.9
BMI, median, IQR	23.6	20.8–27.8
Initial presentation		
Visual, *n*, %	133	33.3
Pyramidal, *n*, %	246	61.7
Sensory, *n*, %	197	49.4
Brainstem, *n*, %	111	27.8
Cerebellar, *n*, %	236	59.1
Bowel and bladder, *n*, %	66	16.5
Cognitive, *n*, %	60	15
Mobility impairment, *n*, %	36	9.3
More than one functional system, *n*, %	273	68.4
EDSS at diagnosis, median, IQR	2.0	1.5–2.5
Current EDSS, median, IQR	3	2.0–3.5
Disease duration, years, median, IQR	11	5–17
Number of relapses, median, IQR	3	2–5
Moderate-efficacy DMT, *n*, %	340	85.2
High-efficacy DMT, *n*, %	59	14.8
DMT more than 1, *n*, %	185	46.4
Switch		
No switch, *n*, %	215	53.9
Horizontal, *n*, %	143	35.8
Vertical, *n*, %	16	4
Both, *n*, %	25	6.3
DMT Duration, years, median, IQR	6	2–10
Time from diagnosis to DMT initiation, median, IQR	2	1–7
PSQI, median, IQR	7	5–9
ISI, median, IQR	5	1–9
ESS, median, IQR	5	3–8
FSS, median, IQR	25	16–42
STOP-Bang, median, IQR	1	1–2
HAM-A, median, IQR	11	5–17

Note: BMI, Body mass index; EDSS, Expanded Disability Status Scale; DMT, Disease-modifying therapies; PSQI, Pittsburgh Sleep Quality Index; ISI, Insomnia Severity Index; ESS, Epworth Sleepiness Scale; FSS, Fatigue Severity Scale; HAM-A, Hamilton Anxiety Rating Scale.

**Table 2:** Internal Consistency of the Questionnaires Used.

Questionnaire	Cronbach’s α	ITC	α If Item Deleted
PSQI	0.78	0.44–0.70	0.71–0.76
ESS	0.73	0.45–0.54	0.67–0.70
FSS	0.95	0.63–0.86	0.95–0.96
ISI	0.88	0.60–0.74	0.85–0.87
HAM-A	0.89	0.49–0.69	0.88–0.89
STOP-Bang	0.45	−0.01–0.36	0.34–0.53

Note: ITC, item–total correlations; PSQI, Pittsburgh Sleep Quality Index; ESS, Epworth Sleepiness Scale; FSS, Fatigue Severity Scale; ISI, Insomnia Severity Index; HAM-A, Hamilton Anxiety Rating Scale.

**Table 3:** Pearson correlations between questionnaire scores.

	PSQI	ISI	FSS	ESS	HAM-A	STOP-Bang
PSQI	—	0.78 **	0.51 **	0.30 **	0.58 **	0.15 *
ISI		—	0.60 **	0.40 **	0.62 **	0.13 *
FSS			—	0.48 **	0.66 **	0.24 **
ESS				—	0.47 **	0.12 *
HAM-A					—	0.19 **
STOP-Bang						—

Note: Values represent Pearson’s r. * *p* < 0.05; ** *p* < 0.001. PSQI, Pittsburgh Sleep Quality Index; ISI, Insomnia Severity Index; FSS, Fatigue Severity Scale; ESS, Epworth Sleepiness Scale; HAM-A, Hamilton Anxiety Rating Scale.

**Table 4:** Characteristics of good vs. poor sleepers.

Variable	Good Sleepers, *N* = 232	Poor Sleepers, *N* = 167	*p*-Value
Age, median (IQR)	39 (34–46)	43 (35–50)	0.02
Female sex, *n* (%)	147 (63.4)	120 (71.9)	0.11
BMI, median (IQR)	23.8 (20.8–27.8)	23.4 (21.0–27.8)	0.94
Initial presentation			
Visual, *n* (%)	75 (32.3)	58 (34.7)	0.39
Pyramidal, *n* (%)	142 (61.2)	104 (62.3)	0.42
Sensory, *n* (%)	112 (48.3)	85 (50.9)	0.39
Brainstem, *n* (%)	66 (28.4)	45 (27)	0.41
Cerebellar, *n* (%)	130 (56)	106 (63.5)	0.17
Bowel and bladder, *n* (%)	32 (13.8)	34 (20.4)	0.11
Cognitive, *n* (%)	30 (12.9)	30 (18.0)	0.21
Mobility impairment, *n* (%)	16 (6.9)	20 (12.0)	0.12
More than one functional system, *n* (%)	157 (67.7)	116 (69.5)	0.79
EDSS at diagnosis, median (IQR)	2.00 (1.5–2.5)	2.00 (1.5–2.5)	0.74
Current EDSS, median (IQR)	3.00 (2.0–3.5)	3.00 (2.5–3.5)	0.27
Disease duration, years, median (IQR)	12 (5–17)	11 (5.5–17)	0.57
Number of relapses, median (IQR)	4 (2–6)	3 (2–5)	0.10
DMT			0.03
Moderate-efficacy DMT, *n* (%)	190 (81.9)	150 (89.8)	
Interferon beta-1a, *n* (%)	35 (15.1)	18 (10.8)	
Interferon beta-1b, *n* (%)	1 (0.4)	2 (1.2)	
Peginterferon beta-1a, *n* (%)	8 (3.4)	3 (1.8)	
Glatiramer acetate, *n* (%)	56 (24.1)	50 (29.9)	
Dimethyl fumarate, *n* (%)	76 (32.8)	66 (39.5)	
Teriflunomide, *n* (%)	14 (6.0)	11 (6.6)	
High-efficacy DMT, *n* (%)	42 (18.1)	17 (10.2)	
Fingolimod, *n* (%)	15 (6.5)	6 (3.6)	
Cladribine, *n* (%)	1 (0.4)	1 (0.6)	
Natalizumab, *n* (%)	4 (1.7)	1 (0.6)	
Ocrelizumab, *n* (%)	19 (8.2)	6 (3.6)	
Ofatumumab, *n* (%)	3 (1.3)	3 (1.8)	
DMT more than 1, *n* (%)	113 (48.7)	72 (43.1)	0.36
Switch			0.14
No switch, *n* (%)	121 (52.2)	94 (56.3)	
Horizontal, *n* (%)	81 (34.9)	62 (37.1)	
Vertical, *n* (%)	10 (4.3)	6 (3.6)	
Both, *n* (%)	20 (8.6)	5 (3.0)	
DMT duration, years, median (IQR)	7 (3–10)	6 (2–11)	0.61
Time from diagnosis to DMT initiation, median (IQR)	2 (1–7)	2 (0–7.5)	0.54
STOP-Bang, median (IQR)	1 (1–2)	2 (1–3)	<0.001
ISI, median (IQR)	3 (0–5)	9 (6–13)	<0.001
FSS, median (IQR)	21 (12–31)	37 (24–51.5)	<0.001
ESS, median (IQR)	4 (2–7)	6 (3.5–9)	<0.001
HAM-A, median (IQR)	7 (3–12)	16 (11–24)	<0.001

Note: *p* for efficacy class from χ^2^ (or Fisher’s exact) test; no hypothesis testing was conducted at the individual-agent level. PSQI, Pittsburgh Sleep Quality Index; ISI, Insomnia Severity Index; ESS, Epworth Sleepiness Scale; FSS, Fatigue Severity Scale, HAM-A, Hamilton Anxiety Rating Scale; DMT, disease-modifying therapy.

**Table 5:** Univariable and multivariable regression of poor sleep.

Variable	Unadjusted			Adjusted		
	RR	95% CI	*p*-Value	RR	95% CI	*p*-Value
Age	1.01	(1.00–1.02)	0.083	1.01	(0.99–1.02)	0.39
Bowel and bladder dysfunction	1.29	(0.99–1.69)	0.063	1.01	(0.77–1.34)	0.92
Mobility impairment	1.37	(1.00–1.88)	0.051	1.23	(0.90–1.70)	0.22
High-efficacy therapy (vs. moderate)	0.65	(0.43–0.99)	0.046	0.80	(0.57–1.12)	0.19
STOP-Bang	1.14	(1.06–1.23)	<0.001	1.02	(0.94–1.12)	0.62
ISI	1.10	(1.08–1.11)	<0.001	1.07	(1.05–1.09)	<0.001
FSS	1.03	(1.02–1.04)	<0.001	1.0	(0.99–1.01)	0.80
ESS	1.07	(1.04–1.09)	<0.001	0.99	(0.96–1.02)	0.46
HAM-A	1.05	(1.04–1.06)	<0.001	1.02	(1.01–1.04)	0.001

Note: RR, Risk ratio; CI, confidence interval; ISI, Insomnia Severity Index; ESS, Epworth Sleepiness Scale; FSS, Fatigue Severity Scale; HAM-A, Hamilton Anxiety Rating Scale. RR for continuous scales (ISI, HAM-A, ESS, FSS, STOP-Bang) are per 1-point increase unless otherwise specified.

## Data Availability

The data presented in this study are available on request from the corresponding author.

## Abbreviations

The following abbreviations are used in this manuscript:

BMI	Body mass index
CI	Confidence interval
CNS	Central nervous system
DMT	Disease-modifying therapies
EDSS	Expanded Disability Status Scale
EPV	Events-per-variable
ESS	Epworth Sleepiness Scale
FSS	Fatigue Severity Scale
HAM-A	Hamilton Anxiety Rating Scale
IFN-β	Interferon-β
ISI	Insomnia Severity Index
IQR	Interquartile range
MS	Multiple sclerosis
PSQI	Pittsburgh Sleep Quality Index
RR	Risk Ratio
RRMS	Relapsing-remitting multiple sclerosis
VIFs	Variance inflation factors
